# Supplementary material for: Postcode Lottery in Healthcare? Findings from the Scottish National Comprehensive Geriatric Assessment in Secondary Care Audit 2019
Source: Healthcare (Basel). 2022 Jan 14;10(1):161. doi: 10.3390/healthcare10010161 (PMC8775440; doi:10.3390/healthcare10010161)
Supplement: Supplementary file 1 [file healthcare-10-00161-s001.zip › Supplementary S1 - SCoOP Steering Group.pdf]

SCoOP Steering Group

Co-Chairs

Prof P K Myint (University of Aberdeen)
Prof G Ellis (NHS[a] Lanarkshire)

Secretary

Dr AIC Donaldson (University of Aberdeen)

Members

Dr L Beveridge - NHS[a] Tayside
Prof C Black - Grampian DASH[b]
Mr S Buchanan - NHS[a] Scotland
Dr J Burns - BGS[c] Scotland
Dr T Byrne - NHS[a] Forth Valley
Dr A Conley - Dumfries & Galloway
Dr A Coull - NHS[a] Lothian
Dr A Einarsson - NHS[a] Shetland
Dr G Hoyle - NHS[a] Grampian
Mrs G Jordan - HIS[d]
Dr A MacDonald - NHS[a] Highland
Dr C McAlpine - BGS[c] Scotland
Dr M McElroy - NHS[a] Glasgow & Clyde
Dr T Quinn - University of Glasgow
Mr G Ramsay - Scottish Surgical Research Group
Prof Sir L Ritchie - University of Aberdeen
Dr S Shenkin - University of Edinburgh
Dr R L Soiza - Scottish Society of Physicians
Dr R Thomas - NHS[a] Fife
Dr A Watt - NHS[a] Ayrshire & Arran
Mrs K Wood - Pharmacy SIG[e]
Dr D MacDonald – Modernising Patient Pathways Programme

[a]National Health Service
[b]Aberdeen Centre for Health Data Science
[c]British Geriatric Society
[d]Health Improvement Scotland
[e]Special Interest Group
