# Supplementary material for: Postcode Lottery in Healthcare? Findings from the Scottish National Comprehensive Geriatric Assessment in Secondary Care Audit 2019
Source: Healthcare (Basel). 2022 Jan 14;10(1):161. doi: 10.3390/healthcare10010161 (PMC8775440; doi:10.3390/healthcare10010161)
Supplement: Supplementary file 1 [file healthcare-10-00161-s001.zip › Supplementary S10 - Physiotherapy V1.0.pdf]

Health Board	Hospital Code	Band 6[a] Physios - frailty specific	Band 5[b] Physios - frailty specific	Which patients do the physiotherapists see?	Total weekday physiot-herapy review time (hours)	Mean physiothe-rapy review time Mon-Fri (hours)	Total weekend physiot-herapy review time	Mean physiot-herapy review time Sat/Sun
C	1	3	0	All patients admitted to acute geriatrics	0	0	0	0
C	2	0	0	Patients referred by medical/nursing staff and those from outside unit	0	0	0	0
I	3	1	0	Patients referred by medical/nursing staff	40	8	10	5
D	4	0	0	n/a	0	0	0	0
G	5	3.6	0	Patients >65 years old who 'screen positive' for Frailty	50	10	20	10
J	6	1	1	Patients that screen positive for frailty	40	8	16	8
F	8	0	0	All patients admitted to acute geriatrics	15	3	6	3
F	7	2	1	All patients admitted to acute geriatrics	55	11	8	4
L	9	0	0	Physios decide on appropriate patients for them to see	43	8.6	0	0
L	11	1	0	All patients admitted to acute geriatrics	25	5	0	0
L	10	1	0	Decide as a frailty team depending on patient presentation	40	8	0	0
L	12	1	0	Patients referred by medical/nursing staff	25	5	0	0
E	24	0	0	Patients referred by medical/nursing staff	20	4	0	0
E	23	0	0	n/a	0	0	0	0
E	21	0.8	0.2	Patients referred by medical/nursing staff	25	5	0	0
E	22	0	0	Patients referred by medical/nursing staff	2	0.4	0	0
K	14	0	0	n/a	0	0	0	0
K	15	1	0	Patients thought able to be discharged straight from receiving unit - referred by acute physician or geriatrician.	39	7.8	0	0
K	13	1	0	Patients referred by medical/nursing staff	35	7	6	3
M	18	1	1	Patients referred by medical/nursing staff	0	0	0	0
M	16	0	0	n/a	0	0	0	0
M	17	2	0	Physios decide on appropriate patients for them to see	40	8	16	8
A	25	0	0.5	Patients referred by medical/nursing staff	30	6	0	0
H	20	1	0	All patients admitted to acute geriatrics	35	7	10	5
H	19	0.5	0.5	Patients referred by medical/nursing staff	40	8	0	0
B	26	0	0	n/a	0	0	0	0
Mean		0.8	0.2		23.0	4.6	3.5	1.8

[a]Specialist physiotherapist
[b]Physiotherapist
