# Supplementary material for: Postcode Lottery in Healthcare? Findings from the Scottish National Comprehensive Geriatric Assessment in Secondary Care Audit 2019
Source: Healthcare (Basel). 2022 Jan 14;10(1):161. doi: 10.3390/healthcare10010161 (PMC8775440; doi:10.3390/healthcare10010161)
Supplement: Supplementary file 1 [file healthcare-10-00161-s001.zip › Supplementary S11 - Occupational Therapy v1.0.pdf]

Hospital Code	Health Board	How many band 6[a] or above occupational therapists are employed specifically to review acute geriatric admissions in 24 hours?	How many band 5[b] occupational therapists are employed specifically to review acute geriatric admissions in 24 hours?	Which patients do the occupational therapists see?	Which patients do the occupational therapists see? Description of 'other'	Total weekly occu-patio-nal thera-py review time	Total occupa-tional therapy review time Mon-Fri	Mean weekday occupa-tional therapy review time	Total weekend occupa-tional therapy review time	Mean weekend occupa-tional therapy review time
1	C	2	3	All patients admitted to acute geriatrics		0	0	0	0	0
2	C	0	0	Those referred by medical/nursing staff		0	0	0	0	0
3	I	1.4	0	Those referred by medical/nursing staff		50	40	8	10	5
4	D	0	0	n/a		0	0	0	0	0

5	G	3	0	other	Patients>65yearsoldwho'screen positive'forFrailty,usingCGAtool	70	50	10	20	10
6	J	2	0	Other	If screened frailty positive then the OTs will see them - this is discussed after the morning handover	56	40	8	16	8
8	F	0	0	All patients admitted to acute geriatrics		24	20	4	2	1
7	F	3	1	All patients admitted to acute geriatrics		58	50	10	8	4
9	L	0	0	OTs decide which require their input		43	43	8.6	0	0
11	L	0.6	0.4	All patients admitted to acute geriatrics		25	25	5	0	0

10	L	1	0	other	Decide as a team on the ground floor which patients they will see there is a cross fertilisation of skills.	40	40	8	0	0
12	L	1	0	Those referred by medical/nursing staff		30	30	6	0	0
24	E			Those referred by medical/nursing staff		20	20	4	0	0
23	E	1	0	Those referred by medical/nursing staff						
21	E	2		Those referred by medical/nursing staff		35	35	7	0	0

22	E	0	0	Those referred by medical/nursing staff		2	2	0.4	0	0
14	K			n/a		0	0	0	0	0
15	K	1	0	Other	Those patients thought able to go home direct from receiving ward.	40	40	8	0	0
13	K	1	0	Those referred by medical/nursing staff		41	35	7	6	3
18	M	1	1	Other	Can also be referred to by acute medics or MOE team	0	0	0	0	0
16	M	0	0	Those referred by medical/nursing staff		0	0	0	0	0

17	M	2		OTs decide which require their input	Others where indicated after geriatrics ward round	56	40	8	16	8
25	A		1	Those referred by medical/nursing staff		40	40	8	0	0
20	H	1		All patients admitted to acute geriatrics		38	30	6	8	4
19	H	1	0	Those referred by medical/nursing staff		35	35	7	0	0

26	B	0	0	n/a		0	0	0	0	0

[a]Specialist occupational therapist

[b]Occupational therapist
