# Supplementary material for: Postcode Lottery in Healthcare? Findings from the Scottish National Comprehensive Geriatric Assessment in Secondary Care Audit 2019
Source: Healthcare (Basel). 2022 Jan 14;10(1):161. doi: 10.3390/healthcare10010161 (PMC8775440; doi:10.3390/healthcare10010161)
Supplement: Supplementary file 1 [file healthcare-10-00161-s001.zip › Supplementary S12 - MDTs v1.0.pdf]

		How many MDTs[a] are there on each day?		Who regularly joins the MDT[a]?													
Health Board	Hospital Code	Monday-Friday	Saturday-Sunday	Consultant	Registrar	Staff Nurse	Specialist Nurse	Auxiliary Nurse	OTs[b]	PTs[c]	Pharmacist	Dietician	Social Work	Discharge Co-ordinator	Hospital@Home Trep	Others	Doyouhave shared patient notes?
C	1	1	0	Y	N	Y	N	N	Y	Y	Y	N	Y	Y	N	Senior charge nurse, Flow manager	N
	2	0	0	N	N	N	N	N	N	N	N	N	N	N	N		Y
I	3	1	0	Y	N	N	Y	N	Y	Y	N	N	N	N	N	Red cross	Y
D	4	0	0	N	N	N	N	N	N	N	N	N	N	N	N		Y
G	5	1	0	Y	N	N	Y	N	Y	Y	N	N	N	Y	N		Y
J	6	≥2	1	Y	Y	Y	Y	N	Y	Y	N	N	N	N	N		Y
F	8	1	0	Y	N	Y	Y	N	Y	Y	N	N	Y	Y	N	District nurse	N
	7	≥2	1	Y	Y	Y	Y	N	Y	Y	N	N	N	Y	N		Y
L	9	1	0	N	N	N	Y	N	Y	Y	N	N	N	N	N		Y
	11	≥2	0	Y	N	Y	Y	N	Y	Y	N	N	N	N	N		N
	10	≥2	1	Y	N	Y	Y	N	Y	Y	N	N	Y	Y	N		N
	12	0	0	N	N	N	N	N	N	N	N	N	N	N	N		N
E	24	1	0	Y	N	Y	N	N	Y	Y	N	N	Y	N	N	Junior doctors and students	N
	23	0	0	N	N	N	N	N	N	N	N	N	N	N	N		N
	21	0	0	N	N	N	N	N	N	N	N	N	N	N	N		N
	22	0	0	N	N	N	N	N	N	N	N	N	N	N	N		N
K	14	1	0	Y	Y	Y	Y	Y	Y	Y	N	N	N	N	N	Junior doctors	Y
	15	1	0	Y	Y	Y	N	N	Y	Y	N	N	N	Y	N		Y
	13	1	0	Y	N	N	Y	N	Y	Y	Y	N	N	N	N		Y
M	18	1	0	Y	N	Y	N	Y	Y	Y	N	N	N	N	N		Y
	16	1	0	Y	Y	Y	N	Y	Y	Y	N	N	Y	Y	N		Y
	17	0	0	N	N	N	N	N	Y	Y	N	N	N	N	N		Y
A	25	0	0	N	N	Y	N	N	Y	Y	N	N	N	N	N		N
H	20	≥2	≥2	Y	N	Y	N	N	Y	Y	Y	N	Y	Y	N		Y
	19	≥2	0	Y	N	Y	Y	N	Y	Y	Y	N	Y	Y	N		N
B	26	0	0	N	N	Y	N	N	Y	Y	Y	N	Y	Y	N		Y

[a]Multidisciplinary Team
[b]Occupational therapist
[c]Physiotherapist
