# Supplementary material for: Postcode Lottery in Healthcare? Findings from the Scottish National Comprehensive Geriatric Assessment in Secondary Care Audit 2019
Source: Healthcare (Basel). 2022 Jan 14;10(1):161. doi: 10.3390/healthcare10010161 (PMC8775440; doi:10.3390/healthcare10010161)
Supplement: Supplementary file 1 [file healthcare-10-00161-s001.zip › Supplementary S2 - audit questions.pdf]

Hospital details

Please complete the survey below.

Thank you!

1) What is the name of the hospital you are completing this survey for? __________________________________

2) Which health board is the hospital located in? __________________________________

Interface - Patient admission process to geriatrics

In this survey our intention is to look at the first 24 hours of admission, and the initial comprehensive geriatric assessment (CGA) that a patient receives. We are keen to understand that process of assessment and service configuration. When addressing the following questions, please consider the first 24 hours of a patient's admission i.e. the first place and timing of CGA specialty assessment.

Subsequent questionnaires will explore downstream inpatient and community services.

Does your hospital have a discrete area/designated beds for acute patient admissions requiring assessment by a geriatrician?

E.g 'Geriatric Assessment Unit' or 'Acute Care of Elderly Unit'

- ◯ Yes
- ◯ No

Do you have any set age cut off for admissions to be accepted into the acute beds in the geriatric medicine department?

- ◯ No age requirement
- ◯ 65 years and over
- ◯ 70 years and over
- ◯ 75 years and over
- ◯ 80 years and over
- ◯ 85 years and over
- ◯ Other

Age criteria for admission to acute geriatrics: ______________________________

Aside from patient age, do you have any set criteria for a patient to qualify for a bed under the care of specialist acute geriatric services?

- ◯ No - all referrals accepted
- ◯ Yes - specific criteria [please detail]

Details of criteria for patient admission to care under acute geriatric medicine department

____

Is the discrete area for acute geriatrics admissions:

- ◯ A number of beds within another general ward
- ◯ A separate assessment unit (daytime operation Mon-Fri)
- ◯ A separate assessment unit (daytime Mon-Sun)
- ◯ A separate assessment unit (open 24/7)
- ◯ Other (Please explain)

Other

____

What do you call this area for acute geriatric admissions? e.g. frailty unit, ACE unit, GAU ______________________________

Where are frail older adults cared for?

- ☐ General medical ward for adults
- ☐ Other (please explain)

Other ______________________________

From the emergency department (ED) how is the care of older patients transferred into that of geriatricians?

- ☐ Direct referral from ED Team
- ☐ Selected by Geriatric Team working within ED
- ☐ Other (Please describe)

Other ______________________________

Does the hospital have an acute medicine department?

- ◯ Yes
- ◯ No

From the acute medicine department, how is the care of older patients transferred into that of geriatricians?

- ☐ Direct referral from Acute Medicine Team
- ☐ Selected by Geriatric Team working in acute medicine
- ☐ Other

Other __________________________________

Can a GP/ primary care refer a patient directly to your service i.e bypass the acute medicine or emergency department?

- ◯ Yes
- ◯ No

Workforce - Staff Involved in CGA

In your hospital how many geriatrician consultant whole time equivalents do you have overall (include all sessions for this question e.g. full-time geriatrician working between geriatrics and general medicine = 1.0 WTE) ______________________________

In your hospital how many geriatrician consultant whole time equivalents dedicated to geriatrics do you have (Please give total as WTE e.g if full time consultant works 50% general medicine and 50% geriatrics = 0.5 WTE) ______________________________

Considering your total consultant geriatrician workforce, how many sessions per week are assigned to acute work (i.e. assessing new patients)? (E.g. 5 consultants working 3 acute sessions per week = 15 sessions weekly) ______________________________

In your hospital how many consultant vacancies (declared and undeclared) are there? (Please give number of vacancies as whole time equivalent e.g. vacancies for one full-time and one part-time consultant = 1.5 WTE) ______________________________

In your hospital how many specialist registrars in geriatric medicine do you have that hold a national training number (NTN)? (Please include those on secondment or parental leave) ______________________________

In your hospital, what is the total number of registrars currently working in geriatric medicine, please give in whole time equivalents (WTE). (E.g 3 full-time registrars and 1 less than full-time registrar at 60% = 3.6 WTE) ______________________________

How many staff grade geriatric medicine specialists do you have in your hospital (please give in WTE)? ______________________________

In your hospital do you have specialist nurses - with enhanced skills in geriatric medicine distinct from bedside nursing? E.g. Complete admission clerking of patients

◯ Yes
◯ No

How many specialist nurses are employed specifically for acute geriatric medicine?
(please answer as whole time equivalent e.g. fulltime is 1.0 WTE) ______________________________

What tasks do the specialist nurses in geriatric medicine perform? ______________________________

Do you have occupational therapist input to assess admissions to geriatric medicine department within the first 24 hours?

◯ Yes
◯ No

Are these OTs:

◯ Linked specifically to the acute geriatric service
◯ Shared with other services (e.g. also cover units)

Do you have physiotherapist input to assess geriatric admissions within the first 24 hours?

◯ Yes
◯ No

Are these PTs:
- ◯ Linked specifically to the acute geriatric service
- ◯ Shared with other services (e.g. also cover other units)

Do you have pharmacists who review the medication of patients within first 24h of admission?
- ◯ Yes
- ◯ No

Are these pharmacists:
- ◯ Linked specifically to the acute geriatric service
- ◯ Shared with other services (e.g. also cover other units)

Do you have social work input to assess geriatric admissions within the first 24 hours?
- ◯ Yes
- ◯ No

Does your hospital have a bed manager or flow co-ordinator that deals specifically with geriatric patient admissions?
- ◯ Yes
- ◯ No

What is the route of access for new patients based on a medical unit that need input from Psychiatry for Older Adults?
- ◯ There is a psychiatrist for older adults based in the admissions setting where they receive Comprehensive Geriatric Assessment that sees all relevant patients
- ◯ There is a referral system to request assessment by psychiatry for older adults and they would normally see referrals on the day of request
- ◯ There is a referral system to request assessment by psychiatry for older adults, the assessment would not normally happen on the day of request unless an emergency
- ◯ There is no access to a psychiatrist for older adults on site

Comprehensive Geriatric Assessment

During what times do consultants actively review new admissions to geriatrics? Please tick all hours that apply.

	7am	8am	9am	10am	11am	12pm	1pm	2pm	3pm	4pm	5pm	6pm	7pm	8pm	9pm	10pm
Monday	☐	☐	☐	☐	☐	☐	☐	☐	☐	☐	☐	☐	☐	☐	☐	☐
Tuesday	☐	☐	☐	☐	☐	☐	☐	☐	☐	☐	☐	☐	☐	☐	☐	☐
Wednesday	☐	☐	☐	☐	☐	☐	☐	☐	☐	☐	☐	☐	☐	☐	☐	☐
Thursday	☐	☐	☐	☐	☐	☐	☐	☐	☐	☐	☐	☐	☐	☐	☐	☐
Friday	☐	☐	☐	☐	☐	☐	☐	☐	☐	☐	☐	☐	☐	☐	☐	☐
Saturday	☐	☐	☐	☐	☐	☐	☐	☐	☐	☐	☐	☐	☐	☐	☐	☐
Sunday	☐	☐	☐	☐	☐	☐	☐	☐	☐	☐	☐	☐	☐	☐	☐	☐

For the purposes of this study, a multi-disciplinary team meeting (MDT) or 'huddle' is defined as a meeting to discuss patient plans with at least 3 of the following in attendance: doctor, nurse, allied health professional (such as OT/PT).

Please indicate how often MDTs/staff huddles occur on each day of the week to discuss patients newly admitted to geriatrics within the first 24h of admission.

	None	One MDT	Two or more MDTs
Monday	○	○	○
Tuesday	○	○	○
Wednesday	○	○	○
Thursday	○	○	○
Friday	○	○	○
Saturday	○	○	○
Sunday	○	○	○

Which of the following members of staff routinely join a multi-disciplinary team meeting to discuss new geriatric patients admitted within the first 24h? (defined as staff member usually attending MDT meeting)

- ☐ Consultant or Staff grade geriatrician
- ☐ Specialist registrars
- ☐ Staff nurse
- ☐ Specialist nurse
- ☐ Auxiliary nurse
- ☐ Occupational therapist
- ☐ Physiotherapist
- ☐ Pharmacist
- ☐ Dietician
- ☐ Social worker
- ☐ Discharge co-ordinator
- ☐ Hospital at home team member
- ☐ Other

Other __________________________________

Do you have shared notes for all healthcare professionals to write in?

- ◯ Yes
- ◯ No

What is the normal pathway for patients requiring hospital stay after initial comprehensive geriatric assessment?

- ☐ Patient stays in same clinical area which also serves as downstream bed base
- ☐ Patient moves to general medical ward but remains under care of consultant geriatrician
- ☐ Patient moves to general medical ward, no longer under the care of a consultant geriatrician
- ☐ Patient moves to downstream ward specifically for geriatric patients
- ☐ Other (please describe)

Other

____

Departmental statistics

Please complete the survey below.

Thank you!

57) Do you know how many patients were admitted to your acute geriatric service in 2017? __________________________________

58) Do you know how many patients were admitted to your acute geriatric service in 2018? __________________________________

59) Do you know the average length of stay on the acute site for admissions to geriatric medicine (please give to nearest number of days)? __________________________________

Occupational Therapists for Acute Geriatrics

Please complete the survey below.

Thank you!

How many Band 6 or above OTs are employed specifically to review acute geriatric admissions within the first 24h? ______________________________

How many Band 5 OTs are employed specifically to review acute geriatric admissions within the first 24h? ______________________________

Which patients do the OTs see?
- ◯ All patients admitted to acute geriatrics
- ◯ The OTs decide which patients require input
- ◯ The OTs only see patients that medical or nursing staff specifically refer to the team
- ◯ Other - please describe

Other ______________________________

At which times are OTs on duty in the ward to review new patients admitted to geriatrics, please select all hours during which an OT is available? (Please only include OTs on duty in the ward, 'on-call' cover is included in a separate question)

	7am	8am	9am	10a	11a	12p	1pm	2pm	3pm	4pm	5pm	6pm	7pm	8pm	9pm
Monday	☐	☐	☐	m	m	m	☐	☐	,	☐	☐	☐	☐	☐	☐
Tuesday	☐	☐	☐	☐	☐	☐	☐	☐	☐	☐	☐	☐	☐	☐	☐
Wednesday	☐	☐	☐	☐	☐	☐	☐	☐	☐	☐	☐	☐	☐	☐	☐
Thursday	☐	☐	☐	☐	☐	☐	☐	☐	☐	☐	☐	☐	☐	☐	☐
Friday	☐	☐	☐	☐	☐	☐	☐	☐	☐	☐	☐	☐	☐	☐	☐
Saturday	☐	☐	☐	☐	☐	☐	☐	☐	☐	☐	☐	☐	☐	☐	☐
Sunday	☐	☐	☐	☐	☐	☐	☐	☐	☐	☐	☐	☐	☐	☐	☐

If you do not have an on duty OT specifically for acute geriatrics out of hours/ at weekends, is there any alternative arrangement? (e.g. OT can be contacted by pager at weekend) Please detail any additional 'on-call' cover not included in table above and the times this is available. ______________________________________

Physiotherapists for Acute Geriatrics

Please complete the survey below.

Thank you!

How many Band 6 or above PTs are employed specifically to review acute geriatric admissions within the first 24h? ______________________________

How many Band 5 PTs are employed specifically to review acute geriatric admissions within the first 24h? ______________________________

Which patients do the PTs see?
- ◯ All patients admitted to acute geriatrics
- ◯ The PTs decide which patients require input
- ◯ The PTs only see patients that medical or nursing staff specifically refer to the team
- ◯ Other - please describe

Other ______________________________

At what times are PTs available to review patients newly admitted to geriatrics, please select all hours that apply. Please only select times when a PT is on duty in the ward/unit - any 'on-call' will be covered in a separate question.

	7am	8am	9am	10a	11a	12p	1pm	2pm	3pm	4pm	5pm	6pm	7pm	8pm	9pm
Monday	☐	☐	☐	m	m	m	☐	☐	,	☐	☐	☐	☐	☐	☐
Tuesday	☐	☐	☐	☐	☐	☐	☐	☐	☐	☐	☐	☐	☐	☐	☐
Wednesday	☐	☐	☐	☐	☐	☐	☐	☐	☐	☐	☐	☐	☐	☐	☐
Thursday	☐	☐	☐	☐	☐	☐	☐	☐	☐	☐	☐	☐	☐	☐	☐
Friday	☐	☐	☐	☐	☐	☐	☐	☐	☐	☐	☐	☐	☐	☐	☐
Saturday	☐	☐	☐	☐	☐	☐	☐	☐	☐	☐	☐	☐	☐	☐	☐
Sunday	☐	☐	☐	☐	☐	☐	☐	☐	☐	☐	☐	☐	☐	☐	☐

If you do not have an on duty PT specifically for acute geriatrics out of hours/ at weekends, is there any alternative arrangement? (e.g. PT can be contacted by pager at weekend) Please detail any additional 'on-call' cover not included in table above and the times this is available. ______________________________

Emergency Department In-Reach

Please complete the survey below.

Thank you!

Do you have a team that can provide a comprehensive geriatric assessment within the Emergency Department?

- ◯ Yes
- ◯ No

Who is involved in this team?

- ☐ Consultant geriatrician
- ☐ Specialist registrar in geriatrics
- ☐ Specialist nurse for geriatrics
- ☐ Occupational therapist
- ☐ Physiotherapist
- ☐ Pharmacist
- ☐ Other (please specify)

Other ______________________________

How are patients that would benefit from a CGA identified?

- ☐ Referral from ED team to 'in-reach' CGA team by bleep/phone
- ☐ 'In-reach' CGA team are based in ED and self-select appropriate patients
- ☐ 'In-reach' CGA team identify potential patients from patient tracking system
- ☐ Other (please describe)

Other ______________________________

During what times are comprehensive geriatric assessments for emergency department patients available from an 'in-reach' team based continuously within the ED?

	7am	8am	9am	10am	11am	12pm	1pm	2pm	3pm	4pm	5pm	6pm	7pm	8pm	9pm	10pm
Monday	◯	◯	◯	◯	◯	◯	◯	◯	◯	◯	◯	◯	◯	◯	◯	◯
Tuesday	◯	◯	◯	◯	◯	◯	◯	◯	◯	◯	◯	◯	◯	◯	◯	◯
Wednesday	◯	◯	◯	◯	◯	◯	◯	◯	◯	◯	◯	◯	◯	◯	◯	◯
Thursday	◯	◯	◯	◯	◯	◯	◯	◯	◯	◯	◯	◯	◯	◯	◯	◯
Friday	◯	◯	◯	◯	◯	◯	◯	◯	◯	◯	◯	◯	◯	◯	◯	◯
Saturday	◯	◯	◯	◯	◯	◯	◯	◯	◯	◯	◯	◯	◯	◯	◯	◯
Sunday	◯	◯	◯	◯	◯	◯	◯	◯	◯	◯	◯	◯	◯	◯	◯	◯

During what times are comprehensive geriatric assessments for emergency department patients available from an on-request 'in-reach' team? i.e. team has to be contacted by bleep/phone by ED staff

	7am	8am	9am	10am	11am	12pm	1pm	2pm	3pm	4pm	5pm	6pm	7pm	8pm	9pm	10pm
Monday	○	○	○	○	○	○	○	○	○	○	○	○	○	○	○	○
Tuesday	○	○	○	○	○	○	○	○	○	○	○	○	○	○	○	○
Wednesday	○	○	○	○	○	○	○	○	○	○	○	○	○	○	○	○
Thursday	○	○	○	○	○	○	○	○	○	○	○	○	○	○	○	○
Friday	○	○	○	○	○	○	○	○	○	○	○	○	○	○	○	○
Saturday	○	○	○	○	○	○	○	○	○	○	○	○	○	○	○	○
Sunday	○	○	○	○	○	○	○	○	○	○	○	○	○	○	○	○

Please describe briefly what the 'in-reach' team do as part of a CGA for an ED patient: ______________________

Linked Services

Please complete the survey below.

Thank you!

While this survey focuses on the early assessment of patients admitted to geriatrics, we wanted to ask a few questions to help understand other facets and demands of the acute service.

This information will also help to inform the detailed survey development for this area in the future.

Does your acute geriatric service have any options for follow-up on discharge?
◯ Yes
◯ No

Please briefly describe the follow up provisions on discharge (e.g. phone-call by community geriatric nurse specialist) and whether this is for every/ selected patients
____

Does your acute geriatric service have any options for assessment of patients at home as a first step or means to avoid admission (i.e. 'hospital at home')
◯ Yes
◯ No

Please briefly describe your 'hospital at home' model and the scope of this service (i.e. patients seen per day)
____

Does your acute geriatric service provide regular scheduled input into orthopaedics?
◯ Yes
◯ No
◯ N/A - no orthopaedic department

Please briefly describe your ortho-geriatric service including days of operation.
____

Does your acute geriatric service provide regular scheduled input into surgical care of older people?
◯ Yes
◯ No
◯ N/A - no surgical unit at this hospital

Please briefly describe your surgical liaison service for older people
____
