# Supplementary material for: Postcode Lottery in Healthcare? Findings from the Scottish National Comprehensive Geriatric Assessment in Secondary Care Audit 2019
Source: Healthcare (Basel). 2022 Jan 14;10(1):161. doi: 10.3390/healthcare10010161 (PMC8775440; doi:10.3390/healthcare10010161)
Supplement: Supplementary file 1 [file healthcare-10-00161-s001.zip › Supplementary S3 - Remote and Rural Questions.pdf]

**Scottish Care of Older People Audit (SCoOP)**

**Follow-up Questionnaire for Remote and Rural Sites**

Thank you for your assistance in providing information about the availability of specialist geriatric services in your hospital. We recognise that you are likely providing several aspects of care that contribute to what is recognised as a 'Comprehensive Geriatric Assessment' and wanted to gather as much information as possible to build up an accurate picture of your service. We would appreciate if you could please answer the following questions to the best of your knowledge.

1. Who normally looks after the medical care of frail patients over 65 years admitted acutely to hospital? E.g Acute medicine consultant, general medical consultant

2. Do you currently have any input from a consultant in geriatric medicine or official means of obtaining advice? eg. Telephone advice from other hospital, locum geriatrician

3. Do you have occupational therapists that see frail, older patients?

4. Do the OTs see all frail older patients, and if not how do they select who they will see e.g. request by nurses/medical team?

5. What days of the week are the OTs available?

6. Do you have physiotherapists that see frail, older patients?

7. Do the PTs see all frail older patients, and if not how do they select who they will see e.g. request by nurses/medical team?

8. What days of the week are the PTs available?

9. Do you have regular meetings about your frail older patients which are attended by a doctor, a nurse, a physiotherapist and an occupational therapist? If so, how often are these meetings scheduled?

Answers to the following questions would be helpful, but not essential if you have no means of obtaining the information.

10. Do you know how many patients over 65 years were admitted in 2017?

11. Do you know how many patients over 65 years were admitted in 2018?
