# Supplementary material for: Postcode Lottery in Healthcare? Findings from the Scottish National Comprehensive Geriatric Assessment in Secondary Care Audit 2019
Source: Healthcare (Basel). 2022 Jan 14;10(1):161. doi: 10.3390/healthcare10010161 (PMC8775440; doi:10.3390/healthcare10010161)
Supplement: Supplementary file 1 [file healthcare-10-00161-s001.zip › Supplementary S4 - Frailty Unit v1.0.pdf]

Health Board	Hospital Code	Unit Name	Where is the discrete area for geriatric acute assessment?	Age criteria for admission	Other criteria for admission
F	8	Acute Care of Elderly Unit (ACE)	Set beds within a general ward	No	Frailty assessment tool from HIS
F	7	Geriatric Assessment Unit (GAU)	Separate assessment unit run 7 days a week	No	Typically over 75 years with frailty syndromes. Exclude patients more appropriately dealt with by subspecialty
L	9	Acute Frailty Unit	Separate assessment unit run 7 days a week	65 years and over	No
L	11	Older Adults Assessment Unit	Separate assessment unit run weekday during daytime	75 years and over	Think Frailty screening tool (minimum of 1 criterion). Functional impairment, significant multiple morbidities, resident in carehome, 4AT, dementia, mobility and polypharmacy.
L	10	Acute receiving unit 4:Department medicine for the elderly	Separate assessment unit run 7 days a week	75 years and over	Frail according to HIS and do not need another specialty. Also over 65 from nursing homes.
K	14	Frailty unit	Set beds within a general ward	65 years and over	Frailty screened, then all ages from care homes.
H	20	Acute Medicine for Elderly (AME) Unit	Separate assessment unit run 7 days a week	No	Frailty screening tool, polypharmacy, mobility, cognition, fall and help with ADLs
