# Supplementary material for: Postcode Lottery in Healthcare? Findings from the Scottish National Comprehensive Geriatric Assessment in Secondary Care Audit 2019
Source: Healthcare (Basel). 2022 Jan 14;10(1):161. doi: 10.3390/healthcare10010161 (PMC8775440; doi:10.3390/healthcare10010161)
Supplement: Supplementary file 1 [file healthcare-10-00161-s001.zip › Supplementary S5 - No Frailty Unit v1.0.pdf]

Health Board	Hospital Code	Where are acutely admitted frail older adults cared for?	Age Criteria for patient to qualify for bed under care of acute geriatrics	Other Criteria for patient to qualify for bed under care of acute geriatrics
C	1	Combined Assessment Unit - for all ages of medical patients	65 years and over	Using the clinical frailty score (5-7) and accepted by Acute Care of the Elderly Practitioners
C	2	General Medical Ward	No	Assessment by Geriatrician or Experienced AHP
I	3	AMU and specific DME wards	65 years and over	Falls and instability, care home resident, delirium or dementia, difficulties with ADLs and polypharmacy
D	4	Care of the Elderly Ward	75 years and over	No
G	5	2 General Medical Wards for frail elderly patients	65 years and over	Frailty screening tool, depending on resident of a carehome, new functional decline, dementia, acute confusion, falls, mobility and fear of falling.
J	6	Ageing & Health ward	No	Screened for frailty
L	12	General Medical Ward	65 years and over	No
E	24	General Medical Ward	No	No
E	23	General medical wards	65 years and over	Falls and instability, Care home residents, Delirium/dementia, Difficulties with ADLs, Polypharmacy
E	21	Medicine for the elderly ward and post-acute ward under the care of a geriatrician.	No	No
E	22	General Medical Ward	No	No
K	15	All patients are admitted to an Emergency medical ward, if appropriate assessed by a Geriatrician and then sent to a Geriatric Assessment ward	65 years and over	No need for subspecialty input
K	13	Can be directly admitted to care of the elderly wards	65 years and over	Frailty - falls, confusion, immobility, multiple co-morbidities and not requiring specialty input. Care home residents. Fractures for non-surgical management
M	18	Acute Medical Assessment Unit	65 years and over	Yes
M	16	Medical Admissions unit +/- General Medical Ward	N/A	N/A
M	17	Acute medical unit	65 years and over	Younger patients with discussion and consultant review (frailty, PD)
A	25	General Medical Ward	No	No
H	19	General Medical Ward	65 years and over	Yes
B	26	General Medical Ward	N/A	N/A
