# Supplementary material for: Postcode Lottery in Healthcare? Findings from the Scottish National Comprehensive Geriatric Assessment in Secondary Care Audit 2019
Source: Healthcare (Basel). 2022 Jan 14;10(1):161. doi: 10.3390/healthcare10010161 (PMC8775440; doi:10.3390/healthcare10010161)
Supplement: Supplementary file 1 [file healthcare-10-00161-s001.zip › Supplementary S6 - Routes into Geriatrics v1.0.pdf]

Health Board	Hospital Name	Referred from ED[a] toGeriatrician led Care	Referred from Acute Medicine to Geriatrician led Care	Referred by Geriatrician based in Acute Medicine to Geriatrician led Care	Direct GP[b] admissions to Geriatrician led care	What is the normal pathway for patients requiring hospital stay after initial CGA[c]?
C	1	Yes	No	Yes	No	Patient stays in same area which also serves as downstream bed base
C	2	No	No	No	No	Patient stays in same area which also serves as downstream bed base
I	3	No	No	Yes	Yes	Downstream ward specifically for geriatric patients
D	4	No	No	No	No	Downstream ward specifically for geriatric patients
G	5	No	No	Yes	Yes	Downstream ward specifically for geriatric patients
J	6	Yes	Yes	No	Yes	Downstream ward specifically for geriatric patients
F	8	Yes	No	No	No	Patient stays in same area which also serves as downstream bed base
F	7	Yes	Yes	No	No	Downstream ward specifically for geriatric patients
L	9	Yes	Yes	No	No	Downstream ward specifically for geriatric patients
L	11	Yes	Yes	No	No	Downstream ward specifically for geriatric patients
L	10	Yes	Yes	No	No	Downstream ward specifically for geriatric patients
L	12	No	Yes	Yes	No	Patient moves to general medical ward, not under care of geriatrician
E	24	No	Yes	No	No	Patient moves to general medical ward, still under care of a geriatrician
E	23	No	No	No	No	Patient stays in same area which also serves as downstream bed base
E	21	No	Yes	No	No	Downstream ward specifically for geriatric patients
K	14	No	No	Yes	No	Downstream ward specifically for geriatric patients
K	15	No	No	Yes	No	Downstream ward specifically for geriatric patients
K	13	No	No	No	No	Downstream ward specifically for geriatric patients
M	18	No	Yes	No	Yes	Various: Patient stays in same area which also serves as downstream bed base; Downstream ward specifically for geriatric patients; Patients are cared for in general medical wards, they receive CGA if frail
M	16	No	No	No	No	Patients are cared for in general medical wards, they receive CGA if frail
M	17	No	Yes	Yes	No	Downstream ward specifically for geriatric patients
H	20	No	No	Yes	No	Downstream ward specifically for geriatric patients
H	19	Yes	yes	Yes	No	Patient moves to general medical ward, still under care of a geriatrician

[a]Emergency department
[b]General Practitioner
[c]Comprehensive Geriatric Assessment
