# Supplementary material for: Postcode Lottery in Healthcare? Findings from the Scottish National Comprehensive Geriatric Assessment in Secondary Care Audit 2019
Source: Healthcare (Basel). 2022 Jan 14;10(1):161. doi: 10.3390/healthcare10010161 (PMC8775440; doi:10.3390/healthcare10010161)
Supplement: Supplementary file 1 [file healthcare-10-00161-s001.zip › Supplementary S7 - Medical and ANP Staffing v1.0.pdf]

		Consultant Geriatrician			Registrar Doctors in Geriatrics		Staff Grades[c] and Specialty Doctors	Geriatric Nurse Specialists
Health Board	Hospital Code	Total WTE[a]	Total sessions per week for acute take	WTE Vacancies	Number NTNs[b]	Total WTE	Total WTE	Total WTE
C	1	3	7	4	1	0	0	0
	2	2.2	0.4	2	0	0	0	4
I	3	5	5	1	1	2	0	1.4
D	4	2.5	0	2	0	0	3	0
G	5	9.8	64.5	1.5	2	1	0.9	3
J	6	13	32	1	3	3	2	0
F	8	1	0.5	1.6	0	0	0	2
	7	11.2	41.75	3	8	6.4	0	4
L	9	17	24	1.6	8	5.8	1.4	4
	11	6.4	5	1	1	0	1.8	3
	10	19	23	1	8	7.4	4.6	5.8
	12	2	2.5	2	0	0	1	2
E	24	0.5	0	5	0	0	0	0
	23	4.75	0	0	0	0	2.6	0
	21	1	0	0	0	0	0	0
	22	0	0	1	0	0	0	0
K	14	10	18	2	3	2	3.1	5
	15	8.5	9	0	2	2	0	3
	13	10	11	0.4	2	0.4	2	4
M	18	11.4	6.5	0	3	3	2.2	9
	16	6.7	2	2	2	1	0.4	1
	17	20	11	1	3	3	6	2
A	25	0	0	0	0	0	0	0
H	20	8	15	1	8	4	6	0
	19	6	10	1	1	2	3	1
B	26	0	0	5	0	0	0	0

[a]Whole Time Equivalent
[b]National Training Number
[c]Staff grade are doctors at least two years into specialty training however are not in a consultant training pathway.
