# Supplementary material for: Postcode Lottery in Healthcare? Findings from the Scottish National Comprehensive Geriatric Assessment in Secondary Care Audit 2019
Source: Healthcare (Basel). 2022 Jan 14;10(1):161. doi: 10.3390/healthcare10010161 (PMC8775440; doi:10.3390/healthcare10010161)
Supplement: Supplementary file 1 [file healthcare-10-00161-s001.zip › Supplementary S8 - Consultant Hours Reviewing New Admissions v1.0.pdf]

Health Board	Hospital Code	Monday-Friday Total (Hours)	Daily Mean (M-F[a])	Weekend Total (Hours)	Daily Mean (S&S[b])
C	1	29	5.8	0	0
	2	8	1.6	4	2
I	3	20	4	0	0
D	4	0	0	0	0
G	5	15	3	0	0
J	6	60	12.5	25	12.5
F	8	15	3	0	0
	7	50	10	22	11
L	9	40	8	16	8
	11	35	7	0	0
	10	60	12	24	12
	12	0	0	0	0
E	24	0	0	0	0
	23	0	0	0	0
	21	0	0	0	0
	22	40	8	0	0
K	14	25	5	14	7
	15	5	1	4	2
	13	27	5.4	6	3
M	18	20	4	2	1
	16	8	1.6	0	0
	17	30	6	8	4
A	25	50	10	20	10
H	20	12	2.4	18	9
	19	40	8	0	0
B	26	0	0	0	0
Mean (hours)		22.7	4.6	6.3	3.1

[a]Monday to Friday
[b]Saturday and Sunday
