# Supplementary material for: Postcode Lottery in Healthcare? Findings from the Scottish National Comprehensive Geriatric Assessment in Secondary Care Audit 2019
Source: Healthcare (Basel). 2022 Jan 14;10(1):161. doi: 10.3390/healthcare10010161 (PMC8775440; doi:10.3390/healthcare10010161)
Supplement: Supplementary file 1 [file healthcare-10-00161-s001.zip › Supplementary S9 - AHP and psych v1.0.pdf]

		Occupational Therapists		Physiotherapists		Pharmacists		Social Work	Bed Management	Old Age Psychiatry
Health Board	Hospital Code	Do they review new patients within 24 hours of admission?	Are they specifically linked to acute geriatrics?	Do they review new patients within 24 hours of admission?	Are they specifically linked to acute geriatrics?	Do they review new patients' medications within 24 hours of admission?	Are they specifically linked to acute geriatrics?	Are they available for input within 24 hours of patient admission?	Is there a bed manager specifically linked to acute geriatrics?	What is route of access for new patients that need specialist assessment/ input?
C	1	yes	linked	yes	linked	yes	yes	yes	no	review within hospital, not same day
	2	no	n/a	yes	shared	yes	no	yes	no	review within hospital, not same day
I	3	yes	linked	yes	linked	yes	no	no	no	review within hospital, not same day
D	4	no	n/a	no	n/a	no	n/a	no	no	review within hospital, not same day
G	5	yes	linked	yes	linked	yes	no	no	no	no access on site
J	6	yes	shared	yes	shared	yes	no	no	no	Review within hospital, not same day
F	8	yes	shared	yes	shared	yes	no	yes	no	same day review
	7	yes	linked	yes	linked	yes	yes	no	no	same day review
L	9	yes	shared	yes	shared	yes	yes	no	no	review within hospital, not same day
	11	yes	linked	yes	linked	no	n/a	no	no	review within hospital, not same day
	10	yes	shared	yes	shared	yes	yes	yes	yes	review within hospital, not same day
	12	no	n/a	no	n/a	no	n/a	no	no	review within hospital, not same day
E	24	yes	shared	yes	shared	no	n/a	no	no	no access on site
	23	no	n/a	no	n/a	no	n/a	no	no	review within hospital, not same day
	21	no	n/a	no	n/a	no	n/a	no	no	no access on site
	22	no	n/a	no	n/a	no	n/a	no	no	review within hospital, not same day
K	14	yes	shared	yes	shared	yes	no	no	no	review within hospital, not same day
	15	yes	shared	yes	shared	yes	no	yes	no	review within hospital, not same day
	13	yes	shared	yes	shared	yes	no	no	no	review within hospital, not same day
M	18	yes	shared	yes	shared	yes	no	no	no	review within hospital, not same day
	16	yes	shared	yes	shared	no	n/a	no	no	review within hospital, not same day
	17	yes	shared	yes	shared	yes	no	no	no	same day review
A	25	no	n/a	no	n/a	yes	no	no	no	review within hospital, not same day
H	20	yes	linked	yes	linked	yes	yes	yes	no	psychiatrist based in admission setting
	19	no	n/a	yes	shared	yes	no	yes	no	same day review
B	26	no	n/a	no	n/a	yes	yes	no	no	review within hospital, not same day
